# Encapsulation of ε-Viniferin into Multi-Lamellar Liposomes: Development of a Rapid, Easy and Cost-Efficient Separation Method to Determine the Encapsulation Efficiency

**DOI:** 10.3390/pharmaceutics13040566

**Published:** 2021-04-16

**Authors:** Pauline Beaumont, Arnaud Courtois, Tristan Richard, Stéphanie Krisa, Chrystel Faure

**Affiliations:** 1 UR Œnologie, MIB, EA 4577, USC 1366 INRAE, ISVV, Université de Bordeaux, 33882 Villenave d’Ornon, France; pauline.beaumont@u-bordeaux.fr (P.B.); arnaud.courtois@u-bordeaux.fr (A.C.); tristan.richard@u-bordeaux.fr (T.R.); stephanie.krisa@u-bordeaux.fr (S.K.); 2 Bordeaux INP, UR Œnologie, MIB, EA 4577, USC 1366 INRAE, 33882 Villenave d’Ornon, France; 3 Centre Antipoison et de Toxicovigilance de Nouvelle Aquitaine, Bâtiment UNDR, CHU de Bordeaux, 33076 Bordeaux, France; 4 Department of Chemistry, Université de Bordeaux, CBMN, UMR 5248, 33600 Pessac, France; 5 Bordeaux INP, CBMN, UMR 5248, 33600 Pessac, France

**Keywords:** encapsulation efficiency, ε-Viniferin, multi-lamellar liposomes, adsorption filtration, polyphenol

## Abstract

Onion-type multi-lamellar liposomes (MLLs), composed of a mixture of phosphatidylcholine and Tween 80, were analyzed for their ability to encapsulate ε-Viniferin (εVin), a resveratrol dimer. Their encapsulation efficiency (EE) was measured by UV-VIS spectroscopy using three different separation methods—ultracentrifugation, size exclusion chromatography, and a more original and advantageous one, based on adsorption filtration. The adsorption filtration method consists indeed of using syringe filters to retain the molecule of interest, and not the liposomes as usually performed. The process is rapid (less than 10 min), easy to handle, and inexpensive in terms of sample amount (around 2 mg of liposomes) and equipment (one syringe filter is required). Whatever the separation method, a similar EE value was determined, validating the proposed method. A total of 80% ± 4% of εVin was found to be encapsulated leading to a 6.1% payload, roughly twice those reported for resveratrol-loaded liposomes. Finally, the release kinetics of εVin from MLLs was followed for a 77 day period, demonstrating a slow release of the polyphenol.

## 1. Introduction

Encapsulation involves the incorporation of molecules, macromolecules, particles or microorganisms in small capsules. This technology offers a broad scope of applications in business sectors as varied as pharmaceuticals, cosmetics, agrochemicals and food industries. Probiotics, antioxidants, minerals, flavoring agents, sweeteners, and nutrients are among the ingredients that have already been encapsulated for food applications [1,2]. In this case, encapsulation has been developed to protect the ingredient from moisture, heat, or other extreme conditions, as well as to impede its interactions with the food matrix and to improve the food organoleptic properties (odor, taste, etc.). Encapsulation is also highly beneficial for drug delivery to improve the efficiency of medical treatments [3,4,5]. Microparticles could offer the possibility to accurately control the release rate of the incorporated drug over periods of hours to months and to target a specific site [3,6,7].

For pharmaceutical drugs, and for functional food ingredients having potential health benefits, encapsulation in liposomes is regarded as a solution to enhance their bioavailability (for oral delivery) and biostability [8,9,10]. In one of our recent papers, onion-type multilamellar liposomes were demonstrated to highly increase the solubility of ε-Viniferin (εVin), a resveratrol dimer, and to decrease its cell toxicity and light sensitivity [11]. This natural polyphenol is found in various edible plants [12], and therefore in human food, especially in grape-derived products [13]. It exhibits antioxidant [14], anti-inflammatory [15], anti-proliferative [16], neuroprotective [17,18], and anti-adipogenic [19] properties, generating a growing interest for pharmaceutical and food industries. However, the few pharmacokinetic studies about εVin have demonstrated a very low bioavailability, mainly due to its poor absorption and high metabolism [20,21].

Similar to most of the bioactive molecules used in pharmaceutical industry, this natural compound is valuable, notably because of the several purification steps conducted after extraction. Consequently, when encapsulating such expensive or seldom molecules, the encapsulation efficiency (EE) has to be as high as possible. EE is defined as the percentage, or fraction, of the bioactive molecule added in the process of manufacture that is associated with the carrier. It quantifies the molecule loading. The release kinetics from liposomes is also an important feature as it defines the amount of free molecule available over time to provide the therapeutic effect [22]. The measurement of EE as well as the determination of molecule release profiles require methods to separate the carriers from their dispersion medium. Separation is typically achieved by filtration [23,24], ultra-centrifugation, or centrifugal ultrafiltration [25]. Other possible routes for removal of non-encapsulated material include dialysis-based methods [26], gel-permeation chromatography, ion-exchange chromatography, and size exclusion chromatography [11]. All these techniques present advantages but also drawbacks. Filtration-based methods in which carriers are retained by the filter are not suitable for tiny or/and deformable carriers such as liposomes [27]. Centrifugation methods may lead to carrier leakage [28]. Chromatography processes diminish product yield by column equilibration which dilutes the final product [29], and are time-consuming, similar to dialysis-based ones. Rapidity is yet an important criterion; it is a prerequisite in industry for screening but also to establish the real-time determination of the amount of free drug in the course of its release.

We developed a new separation method to determine carriers’ EE, based on adsorption filtration, and aimed to simplify and speed up the quantification. The proposed method is inspired by the adsorption by membrane filtration technology currently used in water treatment [30]. It was applied to εVin-loaded multi-lamellar liposomes (MLLs) and compared to classical separation methods (size exclusion chromatography, ultracentrifugation) in terms of EE value and advantages/drawbacks. Finally, by applying the adsorption filtration method to εVin–loaded MLLs, the time-dependent release of the polyphenol was established.

## 2. Materials and Methods

### 2.1. Chemicals and Materials

Methanol, ethanol, acetonitrile, Tween 80, ammonium molybdate tetrahydrate, L-ascorbic acid were purchased from Sigma-Aldrich (St. Louis, MO, USA). Syringe filters with 25 mm diameter and 0.45 µm pore size, unless specified, were used for filtration. Whatman^®^ cellulose acetate and Anapore (alumina-based membrane) filters came from Sigma Aldrich, Clearline^®^, polyvinylidene fluorine (PVDF) from Dominique Dutscher (Brumath, France), polytetrafluoroethylene (PTFE) from Carl Roth, and Nylon and polyethylene sulfone (PES) from Fischer Scientific. Lipoid P75 (soybean lecithins with 68–74% phosphatidylcholine and 7–11% phosphatidylethanolamine) was purchased from Lipoid GmbH (Germany). Potassium phosphate monobasic was from Riedel-de Haën while perchloric acid was from Merck Millipore. εVin was extracted and purified from grape shoot as previously described [31]. Purity (>89%) and structure were confirmed by proton NMR and high-resolution mass spectrometry.

### 2.2. Preparation of Onion-Type MLLs

εVin-loaded onions and plain onions (without εVin) were prepared as previously described [11] with some modifications to improve their encapsulation efficiency based on the work of Crauste-Manciet et al. [32]. Briefly, about 200 mg of εVin (4 wt%), Tween 80 (11.5 wt%) and lipoid P75 (52.5 wt%), the onions components, were first individually solubilized in ethanol before gathering the fractions. Ethanol was then removed by evaporation under N_2_ flux (0.1 bar). Two milliliters of Milli-Q water were added to the evaporated mixture and stored at –80 °C for 24 h. Freeze-drying was then conducted (48 h). The obtained powder was then accurately weighed in a 1 mL vial (68 wt% of the total onion mass) and Milli-Q water (16 wt% of the total onion mass) was added. Shearing of the hydrated mixture was conducted (1 min) using a spatula before adding the other half-part of water (16 wt%). Then, three consecutive cycles of shearing–centrifugation were conducted. Shearing was applied for 5 min as was centrifugation (2000 rpm). Three other cycles of shearing–centrifugation were conducted after storing the preparation for 24 h at 4 °C. The obtained viscous paste is composed of onion-type MLLs in close contact [33]. MLLs can be dispersed in Milli-Q water by gentle agitation using a vortex stirrer (500 rpm). The liposomes’ multi-lamellarity was checked by polarized light microscopy (Olympus, BX51) with x60 and x100 magnifications.

### 2.3. Determination of MLLs Size

MLL average diameter was measured by dynamic light scattering (DLS) using a Vasco particle size analyzer (Cordouan Technologies Ltd., Pessac, France). Three acquisitions of 180 s were recorded on a 1 mg mL^−1^ MLL dispersion using a refractive index of 1.45 and 1.33 for the liposomes and the external aqueous phase, respectively. The average diameter was determined using the cumulant method (intensity). MLL diameter was also measured by static light diffusion using a MasterSizer 2000 apparatus (Malvern) on a 30 mg mL^−1^ MLL dispersion. The same refractive indexes were used for the Mie theory.

### 2.4. Determination of MLLs Charge

The zeta potentials of plain MLLs and εVin-loaded MLLs were measured with a ZetaSizer Nano Series (Malvern) using DTS1070 cells. The dispersion was concentrated at 1 mg mL^−1^ in Milli-Q water. Three measurements of 100 runs were performed.

### 2.5. Phosphorus Assay

To determine the percentage of MLLs retained by a 5 µm-pore sized PVDF filter, a phosphorus assay based on the Rouser protocol was carried out since MLLs were composed of phospholipids [34].

First, a calibration curve was established on KH_2_PO_4_ aqueous solutions with concentrations ranging from 4.4 to 22 µg mL^−1^, which corresponded to phosphorus concentrations ranging from 1 to 5 µg mL^−1^. A linear variation (R^2^ = 0.9978) was found. To be in the linear range of the calibration curve, a 250 µg mL^−1^ dispersion of εVin-loaded MLLs was chosen. One milliliter of this dispersion was passed through a 5 µm-pore sized PVDF filter, the filtrate was poured in silicated glass tubes for freeze-drying (48 h). After water removal, 650 µL of 70% perchloric acid was added and the tubes, on which a marble ball was deposited to prevent perchloric acid evaporation, were heated to 180 °C in a block oven for 30 min. After cooling down in ambient air (approximately 5 min), 500 µL of 25 mg ml^−1^ ammonium molybdate solution and 500 µL of 100 mg mL^−1^ freshly-prepared ascorbic acid solution was added. The tubes were then plunged into boiling water for 10 min before reading the absorbance at 800 nm using Milli-Q water as the reference. A negative control was performed on freeze-dried Milli-Q water. The retention percentage of liposomes, %R_lip_, was deduced from the absorbances measured at 800 nm before and after filtration. The error on %R_lip_ value was deduced from 4 different assays.

### 2.6. UHPLC Analysis

The UHPLC apparatus was an Agilent 1290 Series equipped with an auto sampler module, a binary pump with a degasser, a heater/selector column and a UV-VIS diode array detector (DAD). The column used for εVin analysis was a Zorbax SB-C18 100 mm × 2.1 mm i.d., 1.8 µm column equipped with a 2.1 mm × 5 mm i.d. guard column (Agilent). Solvent A was water and solvent B acetonitrile, both acidified with 0.1% formic acid. The gradient used was as follows: 0–1.7 min, 10% B; 1.7–3.4 min, 10–20% B; 3.4–5.1 min, 20–30% B; 5.1–7.8 min, 30% B; 7.8–8.5 min, 30–35% B; 8.5–11.9 min, 35–60% B; 11.9–15.3 min, 60–100% B; 15.3–17 min, 100% B; 17–17.3 min, 100–10% B, and the injection volume was 2 µL. The quantification was performed with an area under curve of εVin at 320 nm (Bruker Data Analysis 3.2) using a εVin standard curve from 0 to 50 µg mL^−1^ in water:methanol (1:1) (R^2^ = 0.9992).

### 2.7. Spectrophotometric Analysis

The spectrophotometer was an UH5300 Hitachi. The 1 cm-pathlength cells in quartz were used filled with 600 µL of solution. The spectra and absorbances were registered using either water or water:methanol (1:1) solutions for reference. Quantification of εVin was performed at 325 nm using a standard curve from 2.5 to 15 µg mL^−1^ (R^2^ = 0.9982, Supplementary Materials Figure S1).

### 2.8. Size Exclusion Chromatography (SEC)

Separation of free εVin from encapsulated εVin was performed as previously described [11]. An 8 × 0.8 cm column filled with Sephacryl S-300 high resolution gel was used. The column was saturated with 400 µL of a 25 mg mL^−1^ dispersion of plain MLLs. Afterwards, 400 µL of a 25 mg mL^−1^ εVin-loaded MLL was deposited on the gel surface and eluted with water. Fractions (1 mL) were collected and their absorbance was measured at 436 nm to select the MLL-containing fractions. These fractions (1 to 5) were gathered, and MLLs were destructed by adding an equal volume of methanol. The released εVin was quantified by UHPLC analysis. From fraction 9 to 25, methanol was used instead of water for elution. Fractions 16 to 20 containing εVin were checked by the UV signal given at 325 nm. These fractions were gathered before UHPLC analysis to quantify the non-encapsulated εVin.

### 2.9. Ultracentrifugation

A 10 mg mL^−1^ of εVin-loaded onion dispersion was prepared. The total concentration of εVin in this dispersion was checked by UHPLC after MLL destruction by methanol addition. Five hundred microliters of the 10 mg mL^−1^ dispersion was centrifuged (Beckmann Coulter, France) for 1 h, at 100,000× *g*, 4 °C with a strong acceleration and gentle deceleration. Both supernatant (containing the free εVin) and pellet (containing the encapsulated εVin) were analyzed by UHPLC after correct dilution to be in the calibration curve. The pellet was treated with methanol before analysis to disrupt εVin-loaded MLLs.

### 2.10. Retention Percentage of εVin by Filters

To determine the affinity of εVin for different filter membranes, a solution of εVin (40 µg mL^−1^ in water containing 4% methanol) was passed through filters, and the absorbance at 325 nm was measured before and after filtration. The percentage of εVin retention, %R_Vin_ was calculated using the following equation:

%R_vin_ = 100 × (1 ‒ Abs_f_/Abs_i_)

where Abs_f_ and Abs_i_ are the absorbances measured at 325 nm on the filtrate and on the εVin initial solution, respectively. The error on %R_vin_ value was deduced from 4 measurements.

## 3. Results

### 3.1. Multilamellar Liposomes Characterization

**Figure 1:**
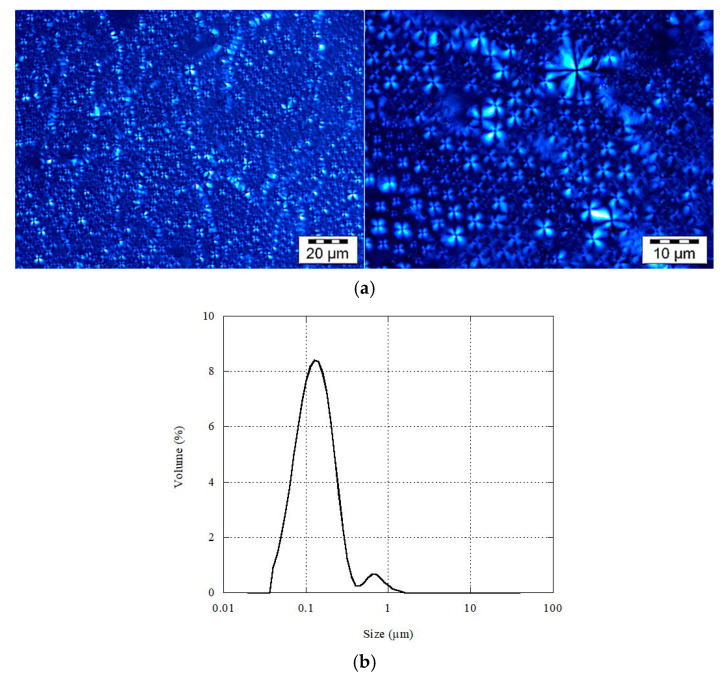
(**a**) Images of multi-lamellar liposomes (MLLs) after shearing observed under polarized light, and (**b**) their size distribution when dispersed in water.

The multi-lamellar character of the εVin-loaded liposomes was checked by optical microscopy under polarized light. As expected, multi-lamellar vesicles were obtained since Maltese crosses, the pattern typical of birefringent MLLs, were observed (Figure 1a). The process we used, i.e., the shearing of a lamellar phase composed of phospholipids and hydrated to its maximal swelling, is indeed well-known to result in the production of MLLs called onions or spherulites [11,32,35,36].

Static light diffusion was conducted on an aqueous dispersion of onions containing 4 wt% εVin. Figure 1b shows their size distribution. A volume representation is given to give evidence for the presence of the largest MLLs (span factor 2.3). Indeed, the R^3^-dependence of the liposome volume, R being the MLLs radius, ensures that large MLLs strongly contribute to the volume so that this type of representation is adapted when their number is low (see Supplementary Materials Figure S2 for number representation). As seen in Figure 1b, MLLs were smaller than 2 µm. Their size was also evaluated by DLS. Their average diameter was 261 ± 5 nm. Their zeta potential was found to be −44 ± 5 mV. The same analyses were conducted on MLLs devoid of εVin—their size was found to be 222 ± 3 nm and their zeta potential −25 ± 5 mV. The change in the zeta potential can be attributed to εVin. Its value measured on εVin-loaded onions was in agreement with previous measurements on onions containing 3 wt% εVin [11]. It was similar to the one reported for phosphatidylcholine-based MLLs containing its monomer, resveratrol [37]. Such an increase in the zeta potential was attributed, in the case of resveratrol, to the aromatic rings of the polyphenol embedded in the membranes [38].

### 3.2. The Adsorption Filtration Method

#### 3.2.1. Description of the Method

**Figure 2:**
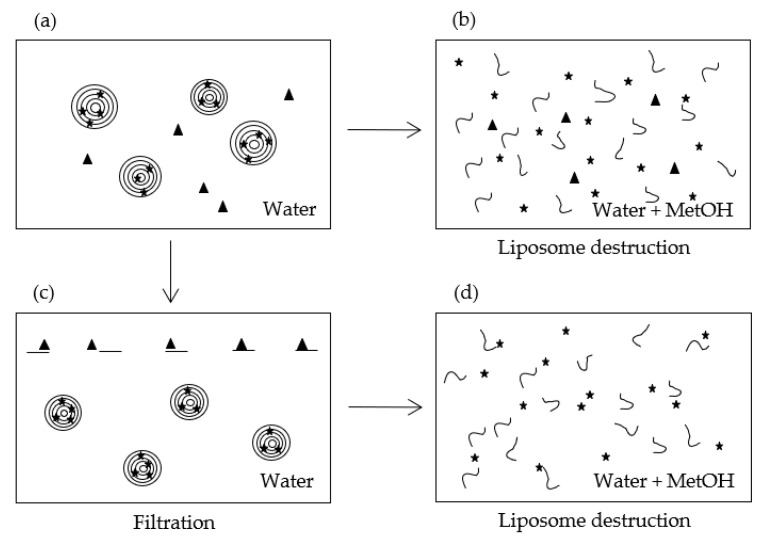
Scheme illustrating the different schemes of the method. (**a**) Dispersion of εVin-loadedMLLs in water, (**b**) destruction of εVin-loadedMLLs, (**c**) filtration of the εVin-loaded MLLs dispersion, (**d**) destruction of the filtrated the εVin-loaded MLLs dispersion. The stars correspond to encapsulated molecules, the triangles to free molecules.

The method requires working with porous membranes so that (i) their pore size is larger than the liposomes’ diameter, and (ii) their composition ensures a high retention of the molecule of interest and a low retention of the liposomes. Hence, in this method, filters are used to retain the material and not the carriers, which is the opposite way of the classical filtering methods. As schemed in Figure 2, part of the liposome dispersion (Figure 2a) is treated in order to break the MLLs and release the encapsulated molecule (Figure 2b) while the other part is filtered (Figure 2c). The filtrate is then also treated to destroy liposomes (Figure 2d). Liposome destruction can be induced by adding either a solvent such as ethanol or methanol, or a surfactant like Triton. In the case of MLLs, Gonzalvez Gomez et al. demonstrated that methanol was much more efficient than Triton [26]. This solvent was then used in this study.

#### 3.2.2. Establishment of EE Equation using UV-VIS Spectrophotometry

UV-VIS spectrophotometry is a rapid and quite cheap technique to assay molecules absorbing in the 200–800 nm range. Polyphenols, such as εVin, belong to this category. When liposomes have a size comparable to the wavelengths of light used in optical spectroscopy, the absorbance signal of the molecule, occurring at λ_max_, is conflated with scattering induced by liposomes. This results in a baseline sloping upwards as the wavelength is lowered [11,39]. This scattering reflects indirectly the size and the volume concentration of the liposomes. Matsuzaki et al. showed that the absorbance measured at 436 nm (turbidity), A_436_, is affected by these two factors [40]. The integrity of MLLs can then be followed by the measurement of A_436_. Once broken by organic solvent addition, light scattering should vanish and the turbidity tends to zero. A_436_ was then registered in the initial dispersion (Figure 2a) to check MLL integrity and after methanol addition (Figure 2b,d) to validate their bursting.

Once liposomes are disrupted, the bioactive molecule is co-solubilized with liposome components. These components could absorb at λ_max_. Their contribution in the whole dispersion after methanol solubilization (Figure 2b) at λ_max_, is given by A_comp_.:

(1) $$A_{\mathrm{comp}.}{=A}_{2}^{\max}-A_{\mathrm{tot}}^{\max}$$

$A_{2}^{\max}$ corresponds to the absorbance measured at λ_max_ on the broken liposomes (Figure 2b), i.e., the signal given by free and released bioactive molecules in the presence of the solubilized liposomes components. $A_{\mathrm{tot}}^{\max}$ is the absorbance given by a solution of bioactive molecules at a concentration equivalent to the one in the liposomes dispersion. $A_{\mathrm{tot}}^{\max}$ is known and is deduced from the standard curve.

Assuming that no liposomes are retained by the filter, the concentration of bioactive compounds in the initial dispersion ${\left[\mathrm{Act}\right]}_{\mathrm{enc}}$ is given by:

(2) $${\left[\mathrm{Act}\right]}_{\mathrm{enc}}{=[\mathrm{Act}]}_{\mathrm{enc}}^{f}=\frac{A_{4}^{\max}-A_{\mathrm{comp}\;}}{\varepsilon l}$$

where ${\left[\mathrm{Act}\right]}_{\mathrm{enc}}^{f}$ is the concentration of the encapsulated bioactive compound in the filtrate, $A_{4}^{\max}$ is the absorbance measured at λ_max_ on the solubilized liposomes after filtration (Figure 2d), l is the cell thickness and ε is the extinction coefficient of the active compound. Let us note that ε was not affected by the solubilized liposomes components in our study.

The encapsulation efficiency, EE, is the percentage of encapsulated active compound relative to the total active used in the dispersion. It is then calculated via the following equation:

(3) $$\mathrm{EE}=100\times \frac{{\left[\mathrm{Act}\right]}_{\mathrm{enc}}}{{\left[\mathrm{Act}\right]}_{\mathrm{tot}}}=100\times \left(\frac{A_{4}^{\max}-A_{\mathrm{comp}}}{A_{\mathrm{tot}}^{\max}}\right)=100\times \left(1-\frac{A_{2}^{\max}-A_{4}^{\max}}{A_{\mathrm{tot}}^{\max}}\right)$$

EE requires then to measure the absorbances at λ_max_ in the MetOH-treated dispersions (Figure 2b,d) and to establish the calibration curve of the bioactive molecule at λ_max_.

In the case where part of the bioactive molecule is retained by filtration, EE must be corrected using Equation (4):

(4) $${\mathrm{EE}}_{c}=100\times \left(1-\frac{A_{2}^{\max}-A_{4}^{\max}}{\%R\times A_{\mathrm{tot}}^{\max}}\right)$$

where %R is the percentage of retention of the bioactive molecule.

In the case where part of the liposomes is retained by the filter, EE is given by the following expression (Supplementary Materials Equations (S1)–(S8)):

(5) $$\mathrm{EE}=100\times \left(\frac{A_{4}^{\max}-A_{\mathrm{comp}.}^{f}}{A_{\mathrm{tot}}^{\max}}\right)\times \frac{100}{\left(100-{\%R}_{\mathrm{lip}}\right)}$$

where %R_lip_ is the percentage of liposomes retained by the filter, and $A_{\mathrm{comp}.}^{f}$ is the contribution at λ_max_ of liposome components after filtration, i.e., in the filtrate.

### 3.3. Determination of εVin EE by Adsorption Filtration

#### 3.3.1. Spectral Analysis of MLLs Components, and εVin

**Figure 3:**
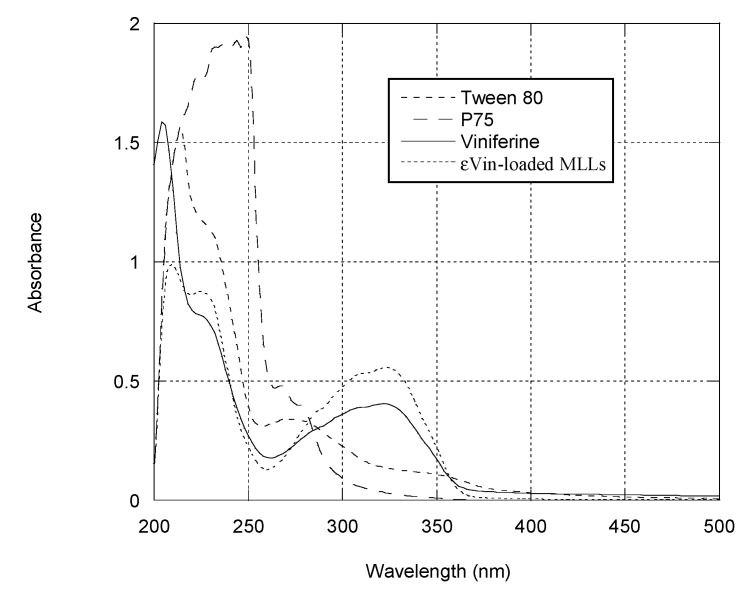
UV-vis spectrum of εVin (10 µg mL^−1^ in MetOH), εVin-loaded MLLs after destruction in MetOH:water (1:1) with εVin concentration of 5 µg mL^−1^, P75 in MetOH:water (1:1) (1 mg mL^−1^), and Tween 80 in MetOH:water (1:1) (8 mg mL^−1^).

Figure 3 collects UV-vis spectra of the different MLL components. As reported in literature, εVin in methanol presents a maximum of absorption at 325 nm [11]. The calibration curve of εVin (Supplementary Materials Figure S1) was established in a mixture of MetOH:water (1:1 *v*:*v*), the solvent in which εVin was co-solubilized with MLL components (Figure 2b,d). After destruction of εVin-loaded MLLs, a peak at 325 nm was observed in the spectrum but its intensity (0.4) is higher than the expected value (0.29) considering εVin concentration (5 µg mL^−1^) and the calibration curve equation. This is due to the contributions of MLL components after MLL destruction as seen in Figure 3—P75 the main component of MLLs also absorbed at 325 nm when solubilized in MetOH:water, as Tween 80 but to a much lesser extent. 

The spectrophotometric behavior of solubilized MLL components (Equation (1)) was then taken into account for the calculation of EE (Equation (3)).

#### 3.3.2. Choice of the Nature of the Syringe Filter Membrane

**Figure 4:**
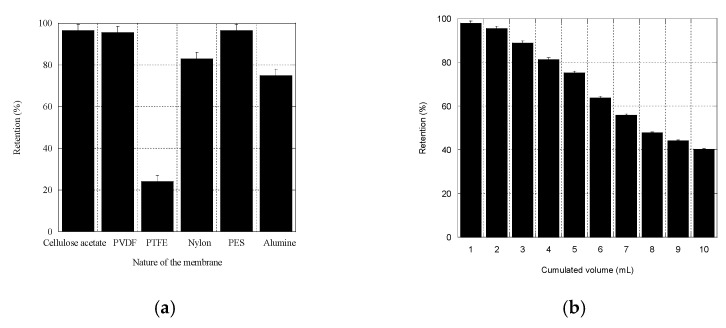
Percentage of retention for a solution of εVin (40 µg mL^−1^) passing through syringe filters. (**a**) Filters differing in the nature of the membrane (filter pore 0.45 µm). (**b**) Polyvinylidene fluorine (PVDF) filter (5 µm pore-sized). Error bars were calculated by measurements on 4 different filters.

The ability of different filters to retain εVin (Figure 4a) was compared by measuring the percentage of retention of εVin, %R_vin_ (Section 2.10).

Among the filters tested (Acetate cellulose, Nylon^®^, PES, PVDF, PTFE), PVDF filters were chosen because their retention efficiency was elevated (97 ± 4%), and because large pore-sized PVDF filters were commercially available. The 5 µm pore-sized PVDF filters were then tested for their limit of saturation. A solution of εVin (40 µg mL^−1^) was filtered through one single filter, and 10 individual fractions (1 mL) were collected in which εVin concentration was quantified by spectrophotometry. Figure 4b shows that %R_Vin_ depended on the filtrated volume. It decreased from 98 ± 3% to 96 ± 3% for 1 to 2 mL and pursued its decrease for larger volumes. Since no more than 2 mL of εVin-loaded MLLs was needed in the adsorption filtration protocol, %R_Vin_ was fixed to 97 ± 4%.

#### 3.3.3. Liposomes Retention by 5 µm Pore-Sized PVDF Filters

Given the onion size distribution (Figure 1b), 5 µm pore-sized filters were chosen. To measure the retention percentage of MLLs, %R_lip_, a phosphorus assay based on the Rouser protocol was conducted before and after filtration on 5 µm-sized PVDF filter. Several rates and MLL concentrations (Supplementary Materials Table S1) were tested to filtrate εVin-loaded MLL dispersions. Whatever the applied rate and the concentration, all the MLLs passed through the filter so that %R_lip_ was 0%. This confirmed that, on one hand, onions were smaller than 5 µm, and, on the other hand, the liposomes had no affinity for the hydrophobic PVDF membrane.

#### 3.3.4. Calculation of the Encapsulation Efficiency

Table 1 gathers the absorbances of the four samples (corresponding to each part of Figure 2) measured at 436 nm (signature of the liposomes, Section 3.2.2) and 325 nm (signature of εVin, Section 3.3.1). From the values measured at 436 nm after methanol treatment, one can conclude that MLLs were indeed disrupted since their values were very low (<0.025). 

The expected value of εVin absorbance, $A_{\mathrm{tot}}^{\max}$ is given by the calibration curve established at λ_max_ = 325 nm (Supplementary Materials Figure S1). The encapsulation efficiency of εVin in onion-type MLLs was calculated to be 80 ± 4% using Equation (3) (Table 2). After correction by the εVin filter retention percentage (%R_εVin_ 97 ± 4%) a final value of 79 ± 4% was found.

### 3.4. Stability of εVin-Loaded MLLs

**Figure 5:**
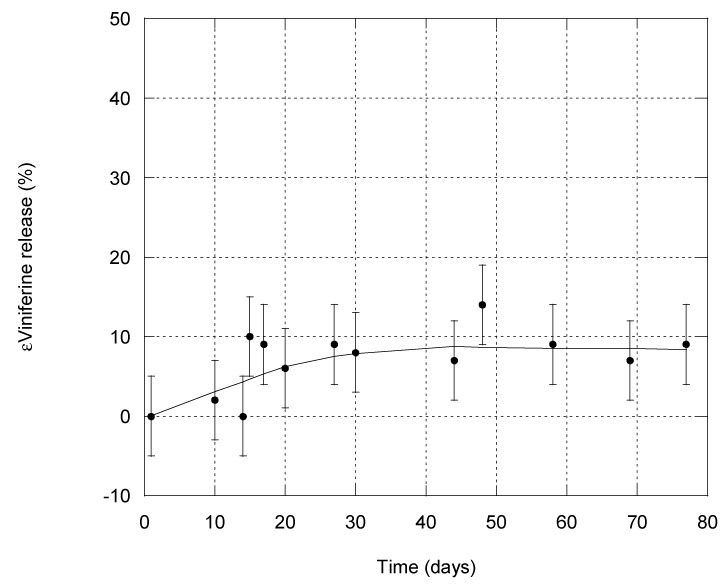
Kinetics of εVin release from onion-type MLLs. The error bar is deduced from three different measurements.

The release of εVin from MLLs was studied for 77 days by repeated quantification of EE using the adsorption filtration separation method. A total of 4 wt% of stilbene was introduced in the composition of MLLs. Before each measurement, the entire dispersion (50 mL at 1 mg mL^−1^) was centrifuged at 500 rpm for 10 min in order to induce precipitation of any εVin that would have leaked from MLLs and formed aggregates (see Section 4.2). This low rate ensured that MLLs did not precipitate. Analyses were then conducted on the supernatant. To quantify EE, A_comp_. was fixed to its initial value of 0.12 (measured on day 1) to remove any bias arising from the removal of part of the free εVin which could have precipitated. Moreover, the value at 436 nm measured on the initial dispersion, $A_{1}^{436}$ (Table 1) was systematically controlled. This value should be constant as long as the liposome structure is maintained (Section 3.2.2). Its value was 0.22 at day 1 and any value differing from 0.22 ± 0.02 was not considered. Microscopic observations were also conducted every week to check that MLL structure was intact; this was the case even after 77 days. The time dependency of EE is reported in Figure 5 and shows that εVin release from MLLs was very slow. After 77 days, 69% of εVin was still encapsulated. As εVin was embedded into the bilayers of MLLs, its release was slow.

### 3.5. Determination of εVin EE by Classical Techniques

In that section, two classical techniques to separate free and encapsulated εVin were used in order to calculate EE—ultra-centrifugation and size exclusion chromatography (SEC).

EE can be expressed by the following equation:

$$\mathrm{EE}=100\times \frac{m_{\mathrm{enc}}}{m_{\mathrm{tot}}}=100\times \frac{\left(m_{\mathrm{tot}}-m_{f}\right)}{m_{\mathrm{tot}}}$$

where m_tot_ is the total mass of εVin in the MLLs dispersion, m_enc_ is the mass of encapsulated εVin, and m_f_ is the mass of free εVin, i.e., the mass of non-encapsulated εVin.

When MLLs were separated by ultracentrifugation, EE was determined in two ways—by the measurement of m_f_ (from the supernatant) and m_i_ (from the sediment). Both values were identical—82 ± 1%.

**Figure 6:**
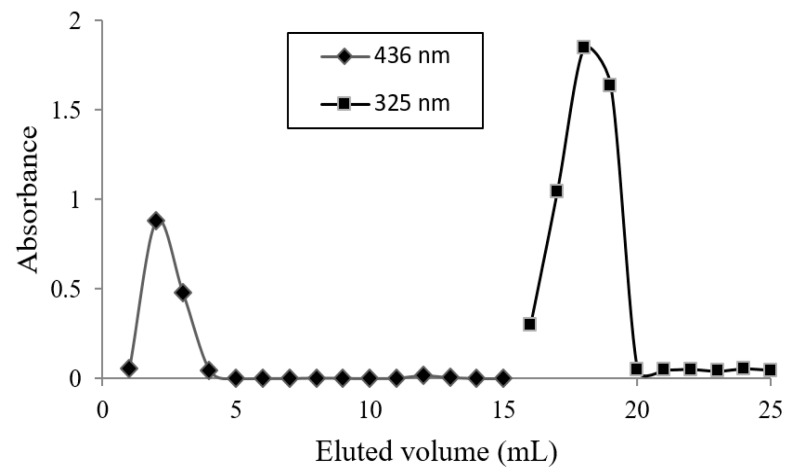
Elution profile of εVin-loaded MLL aqueous suspension. Volume of each fraction—1 mL. Fractions 1 to 8 were eluted with water, and the others with methanol.

The experimental conditions to separate εVin from MLLs by SEC were exposed in a previous study [11]; given εVin has a strong affinity for the two usual SEC gels, Sephadex and Sephacryl, methanol was necessary to elute free εVin. The polyphenol recovery was 98% with this organic solvent. εVin-loaded MLLs were then eluted with water, before collecting εVin with methanol. The absorbance of each 1 mL fraction was measured at 436 and 325 nm and the results are given in Figure 6. MLLs, detected by their 436 nm signal, sorted out of the column from fraction one to four while εVin from fraction 16 to 19. EE was calculated from fractions one to five, i.e., measuring the concentration of encapsulated εVin, by UHPLC. Using the calibration curve, an EE of 78 ± 1% was deduced.

## 4. Discussion

### 4.1. εVin Encapsulation Efficiency and Payload

Compared to our previous study [11], EE was drastically improved—from 58 ± 3% to 80 ± 4%. The same composition was tested but with a higher amount of εVin in this study—4 wt% instead of 3 wt%. This rise in EE is attributed to the modification of the protocol used to prepare εVin-loaded onions. Indeed, based on Crauste-Manciet work on onion-type MLLs, the protocol for lamellar phase hydration was changed. Instead of hydrating with the whole volume of water, it was fractioned in two equal parts and shearing was applied after each water addition (see Section 2.2.). Crauste-Manciet et al. demonstrated that this fractioning increased the proportion of onion-type MLLs as compared to unilamellar vesicles [32]. Considering an EE of 80 ± 4%, the payload, i.e., the bioactive-to-lipid weight percentage [41] reached a value of 6.1% which is equal to the value found by Crauste-Manciet et al. for fisetin, a hydrophobic drug; 6.1%. Moreover, our value was larger than reported values usually below 3% for resveratrol-loaded liposomes [42]. Soo et al. reported, e.g., a loading of 3.08 ± 0.35% in DPPC-Cholesterol liposomes [38].

### 4.2. Comparison of Methods

All the separation methods present a major drawback when working with poorly water-soluble molecules. In this case, aggregation and eventually precipitation of the free molecules could occur. The formation of aggregates into the liposome dispersion would generate errors in the encapsulation ratio deduced from SEC, ultracentrifugation methods and adsorption filtration methods. For SEC, aggregates would elute in the first fractions with liposomes because of their size so that bioactive aggregates would be counted in the encapsulated fraction. For ultracentrifugation, aggregates would precipitate with liposomes introducing the same bias. For the proposed separation process, aggregates affinity for the filter could be different from the single molecules and/or if large enough, these aggregates could clog filter pores so that MLLs could be impeded to cross it. Precipitation of the bioactive is likely to happen in kinetics study because of the release of the biomolecule. For the adsorption filtration method, a solution to avoid this bias is to achieve mild centrifugation (500 rpm, 10 min) before separation to fasten aggregates sedimentation without affecting liposomes dispersion, which is checkable by measuring the dispersion turbidity at 436 nm; it should remain identical to its day-1 value. The supernatant, devoid of aggregates, and still containing the MLLs, can then be analyzed. This was achieved for our kinetics study.

SEC displays several other drawbacks. Firstly, to avoid the retention of part of the bioactive-loaded liposomes in the gel, a saturation of the column gel with usually plain (empty) liposomes has to be achieved. This requires the supplemental preparation of liposomes without bioactive, and also their characterization since their size must be comparable to their bioactive-loaded counterparts, as was the case for us (see Section 3.1). Secondly, samples are diluted by their passage through the column so that the concentration of the bioactive must be large enough to be detectable after elution. A total of 10 mg of εVin-loaded MLLs was used in our case. Thirdly, elution may take a long time (in our case about 1 h). Fourthly, each collected fraction is to be analyzed to detect in which liposomes the bioactive was present, which lengthens the experiment duration. Eventually, in some cases, the molecule displays a strong affinity for the gel so that an organic solvent is employed to elute the molecule of interest; the gel may be impaired and a new column has to be prepared for successive separations. This was the case for εVin, which is why the EE was calculated only from the aqueous fractions, those containing εVin-loaded MLLs. 

Ultracentrifugation also presents several disadvantages. When the density of liposomes is not so different from water, e.g., for uni-lamellar liposomes, high rate and long times (more than 1 h) must be applied to hope to separate them from the free molecule. In a recent work, Gonzalvez Gomez et al. showed that centrifugation was not always successful in separating liposomes from the free molecule, in their case antibiotics, and that high speeds could result in the disruption of liposomes [26]. Moreover, ultracentrifuge is an expensive laboratory experiment.

Compared to SEC and ultracentrifugation, the adsorption filtration method is more rapid (roughly 10 min) allowing its use for “real time” evaluation of release kinetics. It is also very cheap—only one syringe filter was needed and 2 mg of MLLs was sufficient. This implies that a very low amount of bioactive, usually expensive, is necessary. It is adapted to any molecule and type of capsule as long as (i) the filter pore size is much larger than the liposome size, (ii) liposomes display poor affinity for the filters, and (iii) the molecule displays strong affinity for the filters.

Direct filtration, i.e., filtration aimed to retain the liposomes and to let the molecule of interest pass through the filter, obviously presents the same advantages as the proposed method. However, in some cases, it is difficult to find a membrane for which the molecule has no affinity for, like the polyphenol we used (Figure 4a). Moreover, in the case where the liposomes are deformable, some of them could pass through filters, or could be destructured through their passage. In a recent study on MLLs, Touti et al. demonstrated that up to 30% of MLLs composed of a mixture of phosphatidylcholine and Tween 80 went through filters with pores smaller than MLL size, because of their elasticity [36]. Moreover, disruption of MLLs in uni-lamellar liposomes is likely to occur since uni-lamellar liposomes are generally obtained by extrusion, i.e., filtration of MLLs through membranes having calibrated pores smaller than MLL ones [43]. This change in liposome morphology would affect their EE. Filtrating liposomes through large pores, as in the adsorption filtration, avoids these drawbacks.

The proposed method is then adapted for the screening of liposomes formulations but also for the study of fast release kinetics. Nevertheless, as a separation technique, ultracentrifugation should be preferred for large volumes.

## 5. Conclusions

An encapsulation efficiency of 80 ± 4% was obtained for εVin encapsulation in multi-lamellar liposomes. The payload of this resveratrol dimer was then found to be much higher than the reported resveratrol one. EE was measured thanks to the separation of liposomes from the free εVin using two classical techniques and a novel adsorption filtration technique we detailed. This simple, cheap and rapid method could be employed to liposomes but also to any kind of capsule and encapsulated molecule as long as filters are well chosen.

## Figures and Tables

**Table 1:** Values of absorbance measured at 436 nm (MLLs) and 325 nm (εVin) for a 1 mg mL^−1^ dispersion of εVin-loaded MLLs containing 40 µg mL^−1^ of εVin. Filtration was realized with 5 µm pore-sized PVDF filters. MLLs were disrupted by addition of an equal volume of methanol. The standard deviation is ± 0.006.

Figure 2	Dilution	$A_{i}^{436}$	$A_{i}^{325}$
(a) εVin-MLLs whole aqueous dispersion	1	0.221	
(b) εVin-MLLs whole aqueous dispersion plus methanol (1:1)	4	0.023	0.693
(c) Filtered εVin-MLLs aqueous dispersion	1		1.694
(d) Filtered εVin-MLLs aqueous dispersion plus methanol (1:1)	4	0.016	0.578

**Table 2:** Data values to calculate the encapsulation efficiency of εVin in MLLs.

	Value	Notation	Calculation
Percentage of retention of MLLs	0%	%R_lip_	Phosphorus assay
Theoretical A for εVin	0.577	$A_{\mathrm{tot}}^{325}$	Calibration curve
Contribution of lipids and surfactant to the signal given at 325 nm in the whole dispersion	0.116 ± 0.006	A_comp._	Equation (1)
Contribution of εVin to the signal given at 325 nm by the filtrate	0.462 ± 0.006	[Act]^f^_enc_εl	Equation (2)
Encapsulation efficiency	80 ± 4%	EE	Equation (3)
Encapsulation efficiency corrected by %R_Vin_	79 ± 4%	EE_c_	Equation (4)

## Data Availability

The data presented in this study are available within the article.

## Supplementary Materials

The following are available online at https://www.mdpi.com/article/10.3390/pharmaceutics13040566/s1, Figure S1. Validation of the spectrophotometric method and calibration curve of εVin in MetOH:water (1:1). Linearity: y = 0.0556x + 0.0148 with R^2^ = 0.9982. LOD = 0.87 mg/L, LOQ = 2.64 mg/L. Recovery: 103.2% for a concentration of εVin 10µg/mL. Precision: <5% for a concentration of εVin 10 µg/mL. Figure S2. Number representation of the size distribution of ε-viniferin loaded MLLs. Table S1. Effect of MLLs concentration on the retention percentage of 5 µm-sized PVDF filters.

## References

[1] Gibbs B.F., Kermasha S., Alli I., Mulligan C.N. (1999). Encapsulation in the Food Industry: A Review. Int. J. Food Sci. Nutr..

[2] Barroso L., Viegas C., Vieira J., Ferreira-Pêgo C., Costa J., Fonte P. (2021). Lipid-Based Carriers for Food Ingredients Delivery. J. Food Eng..

[3] Tomaro-Duchesneau C., Saha S., Malhotra M., Kahouli I., Prakash S. (2013). Microencapsulation for the Therapeutic Delivery of Drugs, Live Mammalians and Bacterial Cells, and Other Biopharmaceutics: Current Status and Future Directions. J. Pharm..

[4] Singh M.N., Hemant K.S.Y., Ram M., Shivakumar H.G. (2010). Microencapsulation: A Promising Technique for Controlled Drug Delivery. Res. Pharm. Sci..

[5] Pantschwa J.M., Kondiah P.P.D., Choonara Y.E., Marimuthu D., Pillay P. (2020). Nanodrug Delivery Systems for the Treatment of Ovarian Cancer. Cancers.

[6] Vemmer M., Patel A.V. (2013). Review of Encapsulation Methods Suitable for Microbial Biological Control Agents. Biol. Control.

[7] Ahmad R., Srivastava S., Ghosh S., Khare S.K. (2021). Phytochemical Delivery through Nanocarriers: A Review. Colloids Surf. B Biointerfaces.

[8] Lee M.-K. (2020). Liposomes for Enhanced Bioavailability of Water-Insoluble Drugs: In Vivo Evidence and Recent Approaches. Pharmaceutics.

[9] Maritim S., Boulas P., Lin Y. (2021). Comprehensive Analysis of Liposome Formulation Parameters and Their Influence on Encapsulation, Stability and Drug Release in Glibenclamide Liposomes. Int. J. Pharm..

[10] Tian M.-P., Song R.-X., Wang T., Sun M.-J., Liu Y., Chen X.-G. (2018). Inducing Sustained Release and Improving Oral Bioavailability of Curcumin via Chitosan Derivatives-Coated Liposomes. Int. J. Biol. Macromol..

[11] Courtois A., Garcia M., Krisa S., Atgié C., Sauvant P., Richard T., Faure C. (2019). Encapsulation of Viniferin in Onion-Type Multi-Lamellar Liposomes Increases Its Solubility, Its Photo-Stability and Decreases Its Cytotoxicity on Caco-2 Intestinal Cells. Food Funct..

[12] Rivière C., Pawlus A.D., Mérillon J.-M. (2012). Natural Stibenoids: Distribution in the Plant Kingdom and Chemotaxonomic Interest in Vitacea. Nat. Prod. Rep..

[13] El Khawand T., Courtois A., Valls J., Richard T., Krisa S. (2018). A Review of Dietary Stilbenes: Sources and Bioavailability. Phytochem. Rev..

[14] Privat C., Telo J.P., Bernardes-Genisson V., Vieira A., Souchard J.-P., Nepveu F. (2002). Antioxidant Properties of Trans-ε-Viniferin As Compared to Stilbene Derivatives in Aqueous and Nonaqueous Media. J. Agric. Food Chem..

[15] Nassra M., Krisa S., Papastamoulis Y., Kapche G.D., Bisson J., André C., Konsman J.P., Schmitter J.-M., Mérillon J.-M., Waffo-Téguo P. (2013). Inhibitory Activity of Plant Stilbenoids against Nitric Oxide Production by Lipopolysaccharide-Activated Microglia. Planta Med..

[16] Billard C., Izard J.-C., Roman V., Kern C., Mathiot C., Mentz F., Kolb J.-P. (2002). Comparative Antiproliferative and Apoptotic Effects of Resveratrol, ϵ-Viniferin and Vine-Shots Derived Polyphenols (Vineatrols) on Chronic B Lymphocytic Leukemia Cells and Normal Human Lymphocytes. Leuk. Lymphoma.

[17] Caillaud M., Guillard J., Richard D., Milin S., Chassaing D., Paccalin M., Page G., Rioux Bilan A. (2019). Trans ε Viniferin Decreases Amyloid Deposits and Inflammation in a Mouse Transgenic Alzheimer Model. PLoS ONE.

[18] Vion E., Page G., Bourdeaud E., Paccalin M., Guillard J., Rioux Bilan A. (2018). Trans ε-Viniferin Is an Amyloid-β Disaggregating and Anti-Inflammatory Drug in a Mouse Primary Cellular Model of Alzheimer’s Disease. Mol. Cell. Neurosci..

[19] Ohara K., Kusano K., Kitao S., Yanai T., Takata R., Kanauchi O. (2015). ε-Viniferin, a Resveratrol Dimer, Prevents Diet-Induced Obesity in Mice. Biochem. Biophys. Res. Commun..

[20] Courtois A., Atgié C., Marchal A., Hornedo-Ortega R., Lapèze C., Faure C., Richard T., Krisa S. (2018). Tissular Distribution and Metabolism of Trans-ε-Viniferin after Intraperitoneal Injection in Rat. Nutrients.

[21] Kim J., Min J.S., Kim D., Zheng Y.F., Mailar K., Choi W.J., Lee C., Bae S.K. (2017). A Simple and Sensitive Liquid Chromatography–Tandem Mass Spectrometry Method for Trans-ε-Viniferin Quantification in Mouse Plasma and Its Application to a Pharmacokinetic Study in Mice. J. Pharm. Biomed. Anal..

[22] Wallace S.J., Li J., Nation R.L., Boyd B.J. (2012). Drug Release from Nanomedicines: Selection of Appropriate Encapsulation and Release Methodology. Drug Deliv. Transl. Res..

[23] Wagner A., Vorauer-Uhl K., Katinger H. (2002). Liposomes Produced in a Pilot Scale: Production, Purification and Efficiency Aspects. Eur. J. Pharm. Biopharm..

[24] Pattnaik P., Ray T. (2009). Improving Liposome Integrity and Easing Bottlenecks to Production. Pharm. Technol. Eur..

[25] Zhu J., Wang Q., Li H., Zhang H., Zhu Y., Omari-Siaw E., Sun C., Wei Q., Deng W., Yu J. (2018). Galangin-Loaded, Liver Targeting Liposomes: Optimization and Hepatoprotective Efficacy. Drug Deliv. Sci. Technol..

[26] Gonzalez Gomez A., Saifuddin S., Marshall K., Hosseinidoust Z. (2019). Liposomal Nanovesicles for Efficient Encapsulation of Staphylococcal Antibiotics. ACS Omega.

[27] Magenheim B., Levy M.Y., Benita S. (1993). A New in Vitro Rechnique for the Evaluation of Drug Release Profile from Colloidal Carriers—Ultrafiltration Technique at Low Pressure. Int. J. Pharm..

[28] Perkins W.R., Minchey S.R., Ahl P.L., Janoff A.S. (1993). The Determination of Liposome Captured Volume. Chem. Physcis Lipids.

[29] Ruysschaert T., Marque A., Duteyrat J.L., Lesieur S., Winterhalter M., Fournier D. (2005). Liposome Retention in Size Exclusion Chromatography. BMC Biotechnol..

[30] Chaudhary D.S., Vigneswaran S., Ngo H.H., Shim W.G., Moon H. (2003). Biofilter in Water and Wastewater Treatment. Korean J. Chem. Eng..

[31] Biais B., Krisa S., Cluzet S., Da Costa G., Waffo-Teguo P., Mérillon J.-M., Richard T. (2017). Antioxidant and Cytoprotective Activities of Grapevine Stilbenes. J. Agric. Food Chem..

[32] Crauste-Manciet S., Larquet E., Khawand K., Bessodes M., Chabot G.G., Brossard D., Mignet N. (2013). Lipidic Spherulites: Formulation Optimisation by Paired Optical and Cryoelectron Microscopy. Eur. J. Pharm. Biopharm..

[33] Diat O., Roux D., Nallet F. (1993). Effect of Shear on a Lyotropic Lamellar Phase. J. Phys. II.

[34] Rouser G., Fkeischer S., Yamamoto A. (1970). Two Dimensional Thin Layer Chromatographic Separation of Polar Lipids and Determination of Phospholipids by Phosphorus Analysis of Spots. Lipids.

[35] Olea D., Faure C. (2003). Quantitative Study of the Encapsulation of Glucose Oxidase into Multilamellar Vesicles and Its Effect on Enzyme Activity. J. Chem. Phys..

[36] Touti R., Noun M., Guimberteau F., Lecomte S., Faure C. (2020). What Is the Fate of Multi-Lamellar Liposomes of Controlled Size, Charge and Elasticity in Artificial and Animal Skin?. Eur. J. Pharm. Biopharm..

[37] Isailović B.D., Kostić I.T., Zvonar A., Đorđević V.B., Gašperlin M., Nedović V.A., Bugarski B.M. (2013). Resveratrol Loaded Liposomes Produced by Different Techniques. Innov. Food Sci. Emerg. Technol..

[38] Soo E., Thakur S., Qu Z., Jambhrunkar S., Parekh H.S., Popat A. (2016). Enhancing Delivery and Cytotoxicity of Resveratrol through a Dual Nanoencapsulation Approach. J. Colloid Interface Sci..

[39] Dorrington G., Chmel N.P., Norton S.R., Wemyss A.M., Lloyd K., Amarasinghe D.P., Rodger A. (2018). Light Scattering Corrections to Linear Dichroism Spectroscopy for Liposomes in Shear Flow Using Calcein Fluorescence and Modified Rayleigh-Gans-Debye-Mie Scattering Glen. Biophys. Rev..

[40] Matsuzaki K., Murase O., Sugishita K., Yoneyama S., Akada K., Ueha M., Nakamura A., Kobayashi S. (2000). Optical Characterization of Liposomes by Right Angle Light Scattering and Turbidity Measurement. Biochim. Biophys. Acta.

[41] Xu X., Khan M.A., Burgess D.J. (2012). A Quality by Design (QbD) Case Study on Liposomes Containing Hydrophilic API: II. Screening of Critical Variables, and Establishment of Design Space at Laboratory Scale. Int. J. Pharm..

[42] Amri A., Chaumeil J.C., Sfar S., Charrueau C. (2012). Administration of Resveratrol: What Formulation Solutions to Bioavailability Limitations?. J. Control. Release.

[43] Hope M.J., Bally M.B., Webb G., Cullis P.R. (1985). Production of Large Unilamellar Vesicles by a Rapid Extrusion Procedure. Characterization of Size Distribution, Trapped Volume and Ability to Maintain a Membrane Potential. Biochim. Biophys. Acta.

