# Supplementary material for: Encapsulation of ε-Viniferin into Multi-Lamellar Liposomes: Development of a Rapid, Easy and Cost-Efficient Separation Method to Determine the Encapsulation Efficiency
Source: Pharmaceutics. 2021 Apr 16;13(4):566. doi: 10.3390/pharmaceutics13040566 (PMC8073621; doi:10.3390/pharmaceutics13040566)
Supplement: Supplementary file 1 [file pharmaceutics-13-00566-s001.zip › pharmaceutics-1109180-supplementary.pdf]

pharmaceutics

MDPI

Supplementary Materials: Encapsulation of ε-Viniferin into Multi-Lamellar Liposomes: Development of a Rapid, Easy and Cost-Efficient Separation Method to Determine the Encapsulation Efficiency

Pauline Beaumont, Arnaud Courtois, Tristan Richard, Stéphanie Krisa and Chrystel Faure

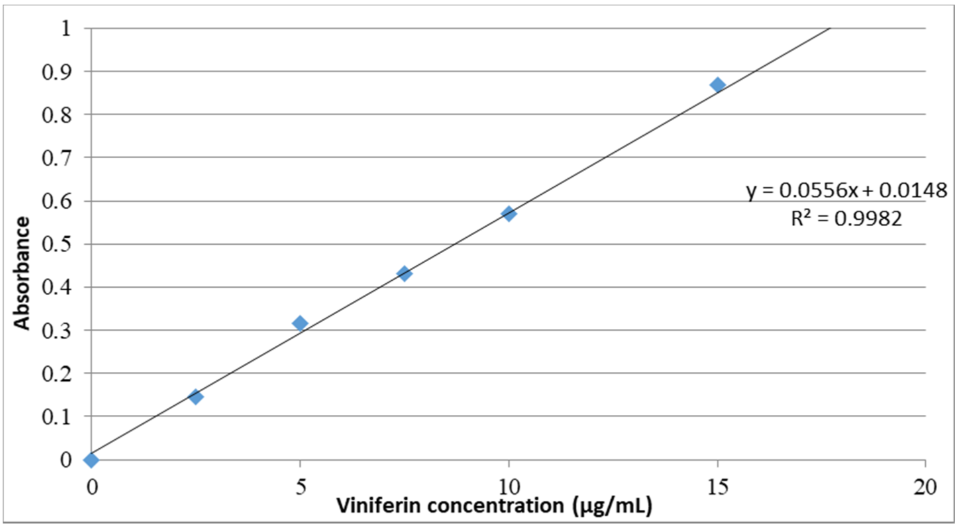

Figure S1. Validation of the spectrophotometric method and calibration curve of εVin in MetOH:water (1:1). Linearity: $y = 0.0556x+0.0148$ with $R^2 = 0.9982$. LOD = 0.87 mg/L LOQ = 2.64 mg/L. Recovery: 103.2% for a concentration of εVin 10μg/mL. Precision: <5% for a concentration of εVin 10 μg/mL.

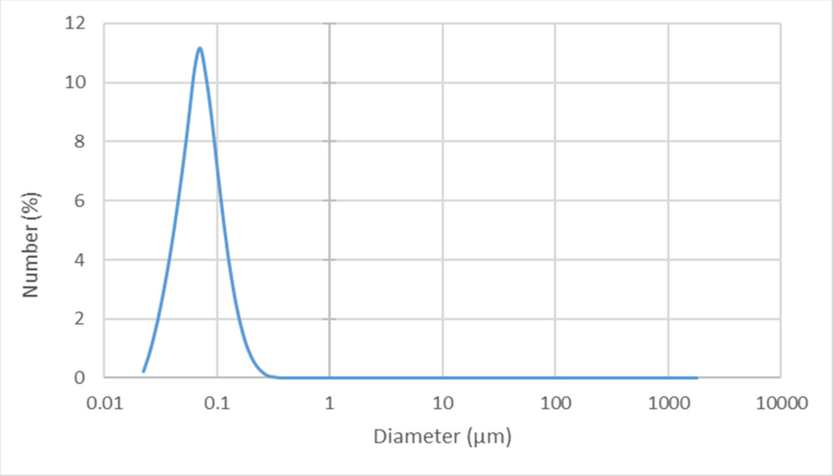

Figure S2. Number representation of the size distribution of ε-viniferin loaded MLLs.

Establishment of EE Equation When Part of Liposomes are Retained by the Filter.

In this case, one can define $\%R_{lip}$, the percentage of liposomes retention by the filter (Equation (1)):

$$\%R_{lip} = 100 \times \frac{[Act]_{enc}^{r}}{[Act]_{enc}} \quad (S1)$$

Knowing $\%R_{lip}$, the percentage of liposomes retention by the filter, one can deduce the contribution at λ_{max} of liposomes' components after filtration, i.e. in the filtrate: $A_{comp.}^{f}$ (scheme 4):

$$A_{comp.}^{f} = \frac{(100 - \%R_{lip})}{100} \times A_{comp.} \quad (S2)$$

The active compound is encapsulated in all liposomes, i.e. those in the filtrate and in the retentate. The concentration of encapsulated bioactive molecules in the initial dispersion is then given by Equation (3):

$$[Act]_{enc} = [Act]_{enc}^{f} + [Act]_{enc}^{r} \quad (S3)$$

with $[Act]_{enc}^{f}$ the concentration of active molecules encapsulated into MLLs present in the filtrate, and $[Act]_{enc}^{r}$, the concentration of molecules encapsulated into the MLLs retained by the membrane.

$[Act]_{enc}^{f}$ can be easily expressed by Equation (4):

$$[Act]_{enc}^{f} = \frac{A_{4}^{\lambda max} - A_{comp.}^{f}}{\varepsilon l} \quad (S4)$$

where $A_{4}^{\lambda max}$ is the absorbance measured at λ_{max} on the solubilized liposomes after filtration (scheme 4), l is the cell thickness and ε is the extinction coefficient of the active compound. Let us note that ε was not affected by the solubilized liposomes components in our study.

Assuming that the bioactive molecule is homogeneously distributed/encapsulated in liposomes, then the percentage of liposomes retention $\%R_{lip}$ is identical to that of the encapsulated compound. Hence:

$$\%R_{lip} = 100 \times \frac{[Act]_{enc}^{r}}{[Act]_{enc}} \quad (S5)$$

Combining Equation 3 and Equation 5 leads to:

$$[Act]_{enc}^{r} = \frac{[Act]_{enc}^{f} \times \%R_{lip}}{100 - \%R_{lip}} \quad (S6)$$

Eventually, one can express $[Act]_{enc}$ from Equation (3), Equation (4) and Equation (6) (Equation (7)):

$$[Act]_{enc} = \left(\frac{A_{4}^{\lambda max} - A_{comp.}^{f}}{\varepsilon l}\right) \times \frac{100}{(100 - \%R_{lip})} \quad (S7)$$

The encapsulation efficiency, EE, is the percentage of encapsulated active compound relative to the total active used in the dispersion. It is then calculated via the following equation (8):

$$EE = 100 \times \frac{[Act]_{enc}}{[Act]_{tot}} = 100 \times \left(\frac{A_{4}^{\lambda max} - A_{comp.}^{f}}{A_{tot}^{\lambda max}}\right) \times \frac{100}{(100 - \%R_{lip})} \quad (S8)$$

Table S1. Effect of MLLs concentration on the retention percentage of 5 μm-sized PVDF filters.

Concentration (mg/mL)	Retention (%)
1	0
10	0
20	0
